# DeepLabV3+ with MobileNetV3 backbone enhancement and multi-level fusion decoder for pepper field weed segmentation

**DOI:** 10.3389/fpls.2026.1877896

**Published:** 2026-07-31

**Authors:** Jun Lu, Fan Wang, Donglin Cao, Tianshu Jin

**Affiliations:** 1 School of Intelligent Manufacturing and Robotics, Shanghai Dianji University, Shanghai, China; 2 Yingtian Agricultural Machinery Technology (Taizhou) Co., Ltd., Taizhou, China; 3 Agricultural and Rural Office, Ningbo, China

**Keywords:** attention mechanism, DeepLabV3+, MobileNetV3, pepper–weed segmentation, semantic segmentation, weed management systems

## Abstract

**Introduction:**

Accurate crop–weed segmentation is critical for precision weed management in pepper fields. However, reliable pixel-level discrimination remains challenging because pepper plants and weeds often share similar green textures under conditions of occlusion, illumination variation, and scale changes, while weed regions are frequently small, fragmented, and spatially dispersed.

**Methods:**

This study proposes an improved DeepLabV3+-based semantic segmentation framework for pepper–weed segmentation. MobileNetV3 was employed as an efficiency-oriented backbone, while Adaptive Activation Fusion and Efficient Channel Attention were integrated to enhance nonlinear feature representation and channel-wise recalibration. A customized atrous spatial pyramid pooling module was designed to capture multiscale contextual information, and an OS = 16 hierarchical decoder was developed to fuse high-, mid-, and low-level features with residual refinement for improved boundary recovery. During training, focal cross-entropy loss and Dice loss were combined with weighted sampling, class-aware cropping, and data augmentation to strengthen the learning of difficult weed regions and improve robustness under complex field conditions.

**Results:**

On the independent pepper-field weed test set, the proposed model achieved an mIoU of 95.87%, recall of 97.98%, a boundary F1-score of 95.88%, a boundary IoU of 92.13%, and an inference speed of 46.86 FPS.

**Discussion:**

The results demonstrate that the proposed framework improves region-level segmentation accuracy, boundary quality, and inference efficiency. These advantages indicate its potential for practical precision weeding applications in pepper fields.

## 1 Introduction

Weeds compete with crops for water, nutrients, light, and space, reducing yield and increasing field-management costs. Accurate crop–weed discrimination is therefore essential for site-specific weed management, including targeted spraying, mechanical weeding, and robotic field operations. In pepper production, pixel-level segmentation can reduce unnecessary herbicide use and improve intervention accuracy, but remains challenging because pepper and weed plants often share similar green textures and irregular leaf shapes under variable illumination, occlusion, cluttered backgrounds, and scale changes.

Computer vision and machine-learning methods have supported crop–weed recognition under field conditions, although their performance is often affected by illumination variation, complex backgrounds, plant morphology, and occlusion (Ashraf and Khan, 2020; Al-Badri et al., 2022; Garibaldi-Márquez et al., 2025; Bhandari and Etienne, 2026). Semantic segmentation provides pixel-level localization and is therefore more suitable than image-level classification or object detection for precision weeding. Early pixel-wise studies demonstrated the feasibility of dense crop–weed discrimination, while subsequent encoder–decoder networks improved spatial-detail recovery and boundary prediction (Dyrmann et al., 2016; Khan et al., 2020; Janneh et al., 2023; Joy et al., 2026). Classical segmentation models, including FCN, U-Net, SegNet, PSPNet, DeepLabv3, and DeepLabV3+, improved dense prediction through encoder–decoder structures, pyramid pooling, atrous convolution, and multiscale context aggregation (Long et al., 2015; Ronneberger et al., 2015; Badrinarayanan et al., 2017; Chen et al., 2017; Zhao et al., 2017; Chen et al., 2018). However, many such models require substantial computation, which limits their use on field robots and edge devices.

Lightweight segmentation networks and efficient backbones have been developed to improve deployment efficiency through real-time architectures, depthwise separable convolution, bilateral feature aggregation, and hardware-aware design (Paszke et al., 2016; Romera et al., 2018; Zhao et al., 2018; Sandler et al., 2018; Howard et al., 2019; Yu et al., 2018, Yu et al., 2021; Xu et al., 2023). Nevertheless, reduced model capacity can weaken the recovery of small weed regions, occluded areas, and ambiguous crop–weed boundaries. Recent agricultural studies have incorporated lightweight backbones, multiscale fusion, attention mechanisms, and CNN–Transformer hybrid structures for weed segmentation in maize, cassava, sugarcane, and UAV-based scenarios (Janneh et al., 2023; Habib et al., 2024; Fu et al., 2025; Lu et al., 2025; Sun et al., 2025; Zi et al., 2025). Hu et al. (2025) improved crop detection under visually similar crop appearances and complex boundaries through texture enhancement and multi-layer attention fusion, whereas Sanches et al. (2026) emphasized that mobile weed-recognition systems should be evaluated not only by accuracy but also by latency, memory consumption, and hardware-tier adaptability.

Pepper-field weed segmentation presents agronomic and visual challenges distinct from those in maize, cassava, and sugarcane fields. Pepper plants typically form relatively low, branched canopies with leaves extending close to the soil surface, while small weed seedlings often occur near stems or beneath overlapping foliage. These conditions increase crop–weed boundary ambiguity and reduce the reliability of directly transferring generic crop–weed segmentation models. In the present dataset, pepper and weed pixels accounted for 74.35% and 25.65% of valid labeled pixels, respectively, corresponding to a moderate pepper-to-weed ratio of approximately 2.90:1. However, the primary difficulty arises from the local spatial characteristics of weed regions, which are often small, fragmented, unevenly distributed, and partially occluded by pepper foliage. Although recent methods have improved multi-level feature fusion and lightweight inference, including Janneh et al. (2023) and Fu et al. (2025), they do not explicitly address the combined challenges of reduced representation capacity in lightweight backbones, fragmented weed recovery, ambiguous pepper–weed boundaries, uneven local weed distribution, and efficient deployment under pepper-field-specific conditions.

To address these challenges, this study proposes an improved DeepLabV3+-based semantic segmentation framework for pepper-field weed segmentation. MobileNetV3 is used as an efficiency-oriented backbone, while Adaptive Activation Fusion and Efficient Channel Attention enhance nonlinear representation and channel-wise recalibration. A customized atrous spatial pyramid pooling module aggregates multiscale context, and a hierarchical decoder integrates high-, mid-, and low-level features with residual refinement to improve spatial-detail recovery and boundary localization. Focal Cross-Entropy and Dice loss are combined with targeted sampling and class-aware training strategies to strengthen learning for difficult weed regions. The framework is evaluated against representative mainstream, lightweight, and Transformer-based segmentation models, together with ablation experiments on the proposed modules, decoder variants, loss functions, and class-weight configurations.

## 2 Materials and methods

### 2.1 Dataset acquisition, annotation, and partitioning

The pepper–weed dataset was collected from a pepper cultivation base in Tangbei Village, Shuyuan Town, Pudong New Area, Shanghai, China (121.871110° E, 30.982397° N). Images were captured using an iPhone 15 Pro Max at approximately 50 cm above the ground from nadir and oblique views. The field was planted with a screw pepper cultivar (Capsicum annuum L.) at a row spacing of 40 cm and a plant spacing of 40 cm, corresponding to approximately 62,500 plants ha^-1^. Large crabgrass (*Digitaria sanguinalis*) and common purslane (Portulaca oleracea L.) were the dominant field-observed weeds. Images were acquired on November 23, 2025, during the vegetative stage of pepper development, across midday and late-afternoon periods under sunny and rainy conditions. The dataset included variations in illumination, plant distribution, viewing angle, and local occlusion.

**Figure 1:**
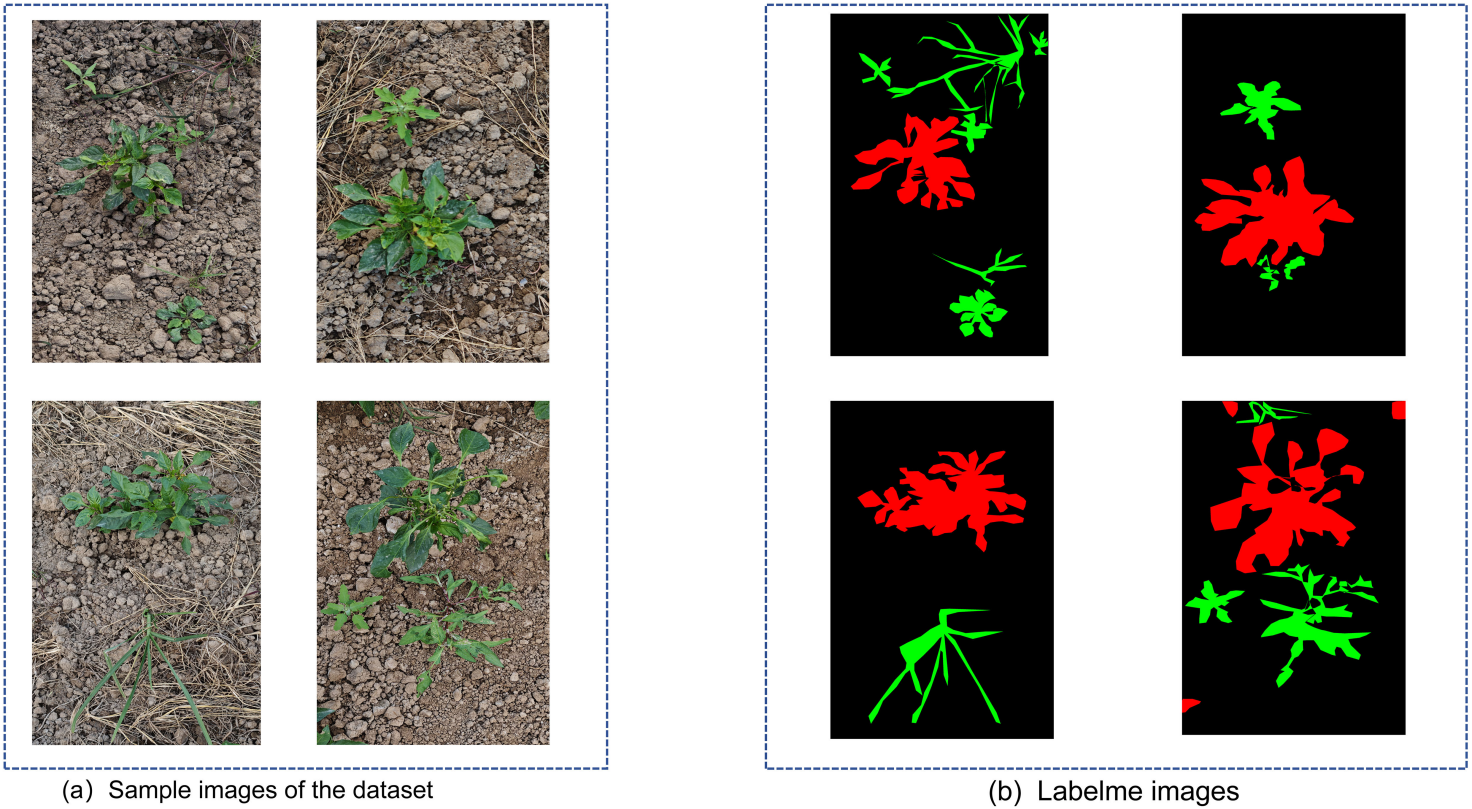
RGB images and labels. **(a)** Sample images; **(b)** Label images.

A total of 2,198 RGB images were used, including 1,978 original images and 220 additional images collected as an independent internal test set. Pixel-level masks were manually created using LabelMe (Russell et al., 2008), with values of 0, 1, and 255 representing pepper, weed, and ignored background pixels, respectively. Ignored pixels were excluded from loss calculation and metric evaluation. The original images were divided into 1,663 training images and 315 validation images. The training set was used for optimization, the validation set for model selection and hyperparameter adjustment, and the internal test set for final evaluation, model comparison, and ablation analysis. All images were acquired as independent still photographs rather than frames extracted from continuous video. The original 1,978 images were randomly divided at the individual-image level into the training and validation subsets. Data collection was organized in separate batches at approximately 15-day intervals, with each batch covering different field plots. To prevent spatial leakage during final evaluation, the 220-image internal test set was collected from field plots that did not overlap with those represented in the training and validation subsets. When multiple views of the same plant were available, they were assigned to a single subset and were not split across the training, validation, and internal test sets. Therefore, the internal test set was not constructed from identical field regions, adjacent capture sequences, or temporally neighboring image sequences of the training and validation data. The task was formulated as two-class semantic segmentation of pepper and weed regions, with background pixels ignored. Representative images and annotations are shown in **Figure 1**.

For cross-location validation, an external test set of 200 RGB images was collected from a pepper field in Yangtian Town, Qingyang County, Chizhou, Anhui Province, China (30.56984° N, 117.94207° E). The field was planted with a screw pepper cultivar (Capsicum annuum L.) at 60 cm × 40 cm spacing, corresponding to approximately 41,667 plants ha^-1^. Large crabgrass (Digitaria sanguinalis) and common purslane (Portulaca oleracea L.) were the dominant observed weeds. Images were acquired on July 4, 2026, during the mature-fruit stage under overcast and rainy conditions at midday. They were annotated using the same protocol and class definitions as the Shanghai dataset and were excluded from training, validation, checkpoint selection, hyperparameter tuning, and fine-tuning.

### 2.2 Data preprocessing and targeted weed-region sampling

All images and masks were processed at 640 × 640 pixels. Images were converted from BGR to RGB and normalized using the ImageNet mean [0.485, 0.456, 0.406] (Deng et al., 2009) and standard deviation [0.229, 0.224, 0.225] to stabilize training and retain compatibility with ImageNet-pretrained backbones. During training, class-aware cropping was applied with a probability of 0.5 to retain informative foreground, particularly weed-containing regions. Crop windows were expanded by a factor of 1.3, with a minimum size of 32 pixels and a random center perturbation of up to 10% of the window size; images and masks were then resized to 640 × 640 pixels.

**Figure 2:**
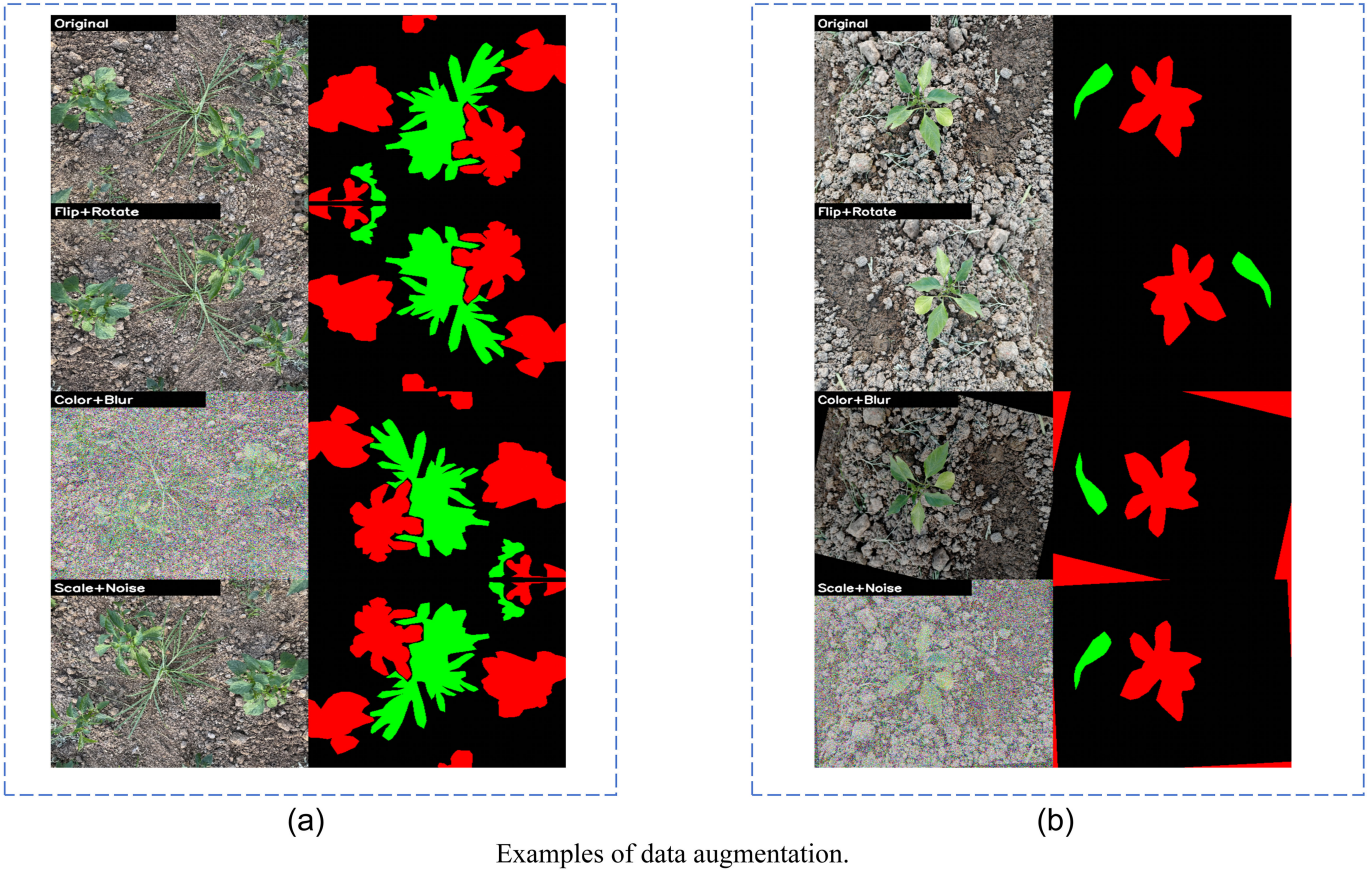
Examples of data augmentation. **(a)** Augmentation results for a representative image with relatively dense pepper and weed regions; **(b)** augmentation results for a representative image with relatively sparse pepper and weed regions. Each panel shows the original image and its segmentation mask, followed by the results of Flip+Rotate, Color+Blur, and Scale+Noise augmentation.

Data augmentation included horizontal and vertical flipping (p = 0.5 each); brightness and contrast jittering (p = 0.7; contrast U(0.7, 1.3); brightness offset U(−30, +30) in uint8 units); and random rotation. For samples containing pepper pixels, rotation was applied with p = 0.7 and angles sampled from U(−30°, +30°); for samples without pepper pixels, it was applied with p = 0.3 and angles sampled from U(−15°, +15°). Random scaling and resizing (p = 0.6; U(0.8, 1.2)), Gaussian blur (p = 0.3; kernel size 3 or 5), HSV jittering (p = 0.5; hue U(−10, +10) on the OpenCV 0–180 scale; saturation and value U(0.8, 1.2)), and additive Gaussian noise (p = 0.2; mean 0, standard deviation 5 in uint8 units) were also applied. Geometric transformations were applied consistently to images and masks, whereas photometric transformations were applied only to images. Examples are shown in **Figure 2**.

Pepper and weed pixels accounted for 74.35% and 25.65% of valid labeled pixels, respectively, yielding a moderate pepper-to-weed ratio of approximately 2.90:1. However, task difficulty was driven mainly by the local distribution of weeds, which were often small, fragmented, unevenly distributed, and partly occluded by pepper leaves. Weighted random sampling therefore assigned higher probabilities to images with relatively larger weed-pixel proportions. Combined with class-aware cropping and the hybrid loss, this strategy increased exposure to difficult weed regions during optimization.

### 2.3 Proposed segmentation framework

#### 2.3.1 Overall architecture

**Figure 3:**
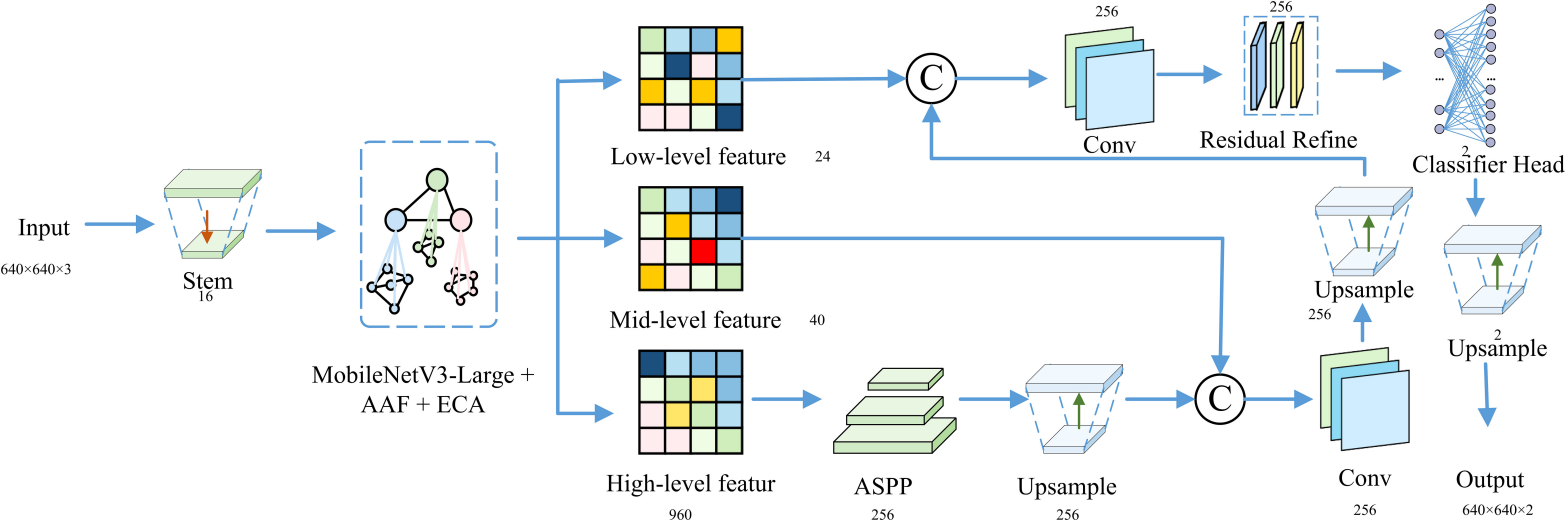
Overall framework of the proposed network.

The proposed model is an improved DeepLabV3+-based encoder–decoder framework for pepper-field weed segmentation (Chen et al., 2018). As shown in **Figure 3**, it consists of a MobileNetV3 backbone enhanced with Adaptive Activation Fusion (AAF) and Efficient Channel Attention (ECA), a customized atrous spatial pyramid pooling (ASPP) module, and an OS = 16 hierarchical decoder with residual refinement. These components were designed to strengthen nonlinear representation, multiscale context aggregation, and boundary recovery in complex field scenes.

Given an input image, the backbone extracts low-, mid-, and high-level features. The high-level feature map is processed by ASPP, and the decoder progressively fuses high-, mid-, and low-level features. Residual refinement is applied after each fusion stage, and the resulting logits are upsampled to the input resolution to generate the final segmentation map.

#### 2.3.2 AAF- and ECA-enhanced MobileNetV3 encoder

**Figure 4:**
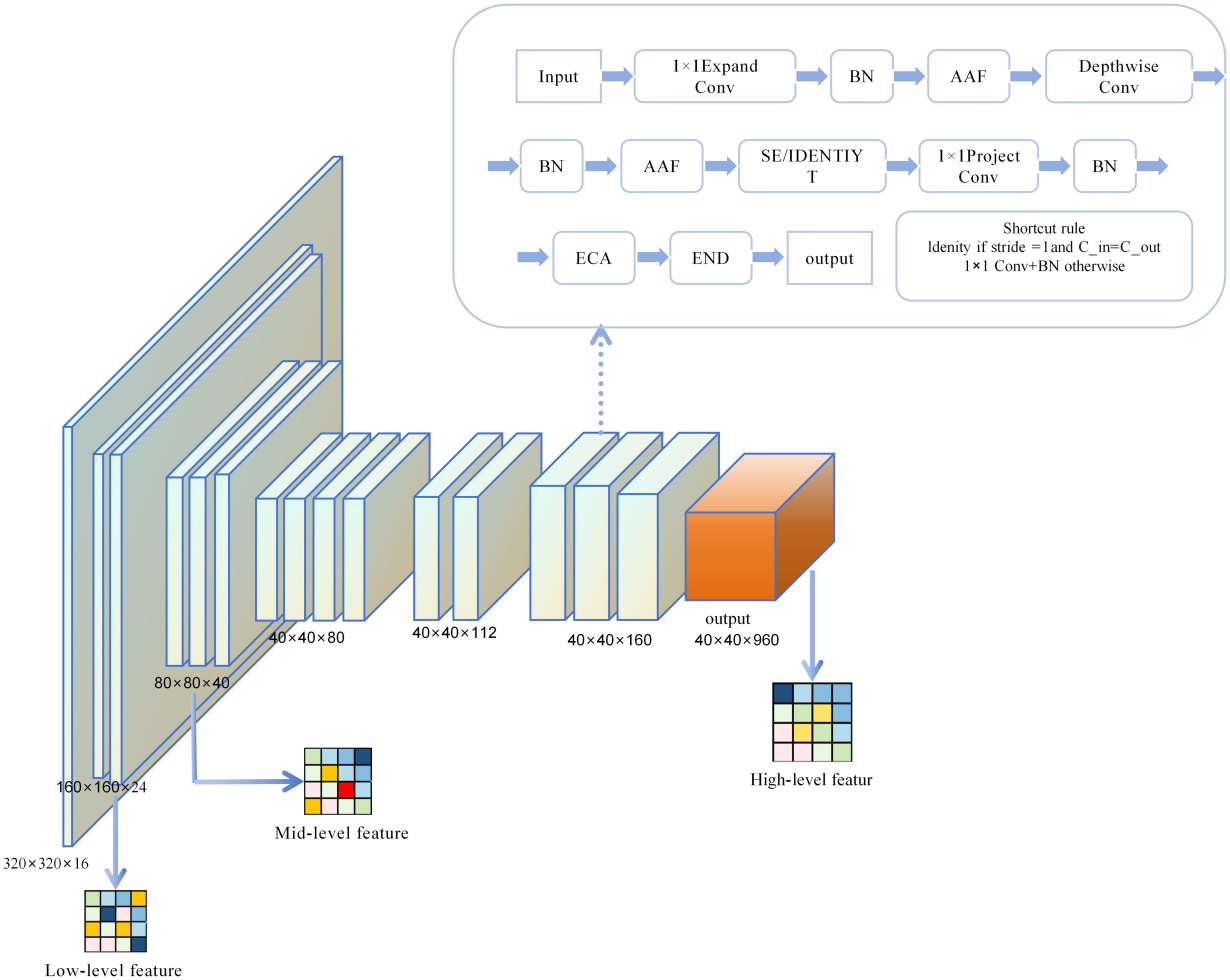
MobileNetV3-based backbone enhanced with AAF and ECA.

**Figure 5:**
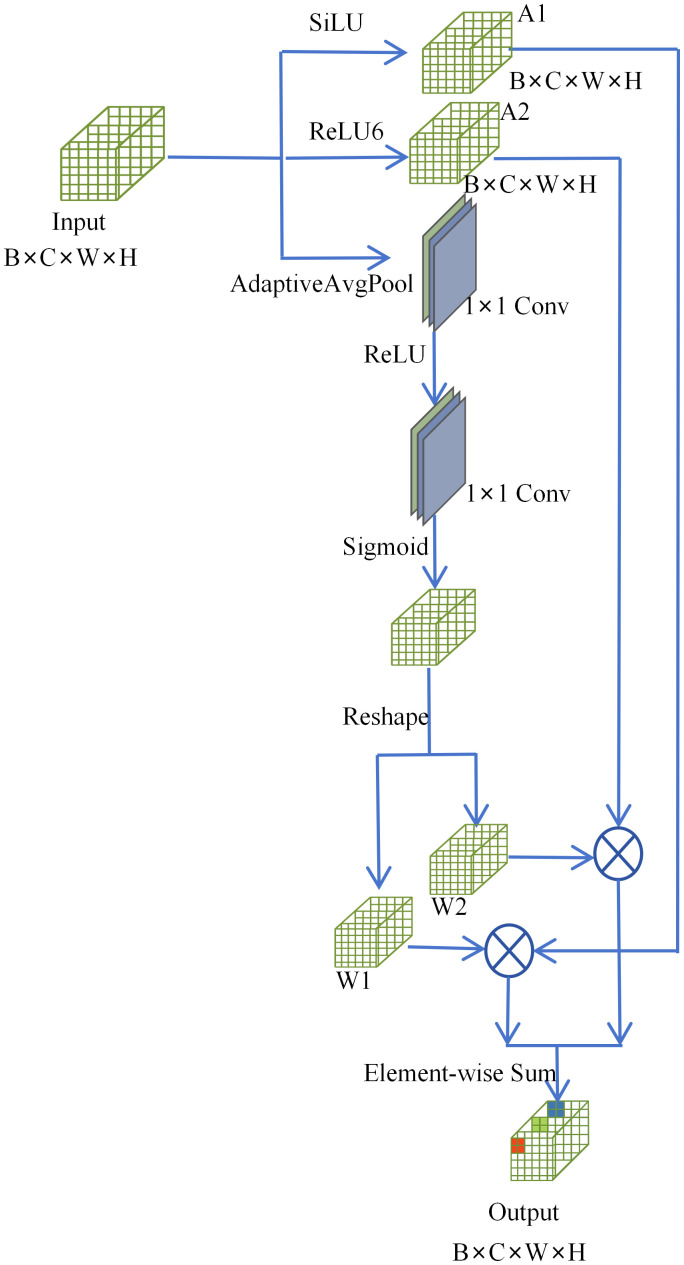
Structure of the adaptive activation fusion (AAF) module.

**Figure 6:**
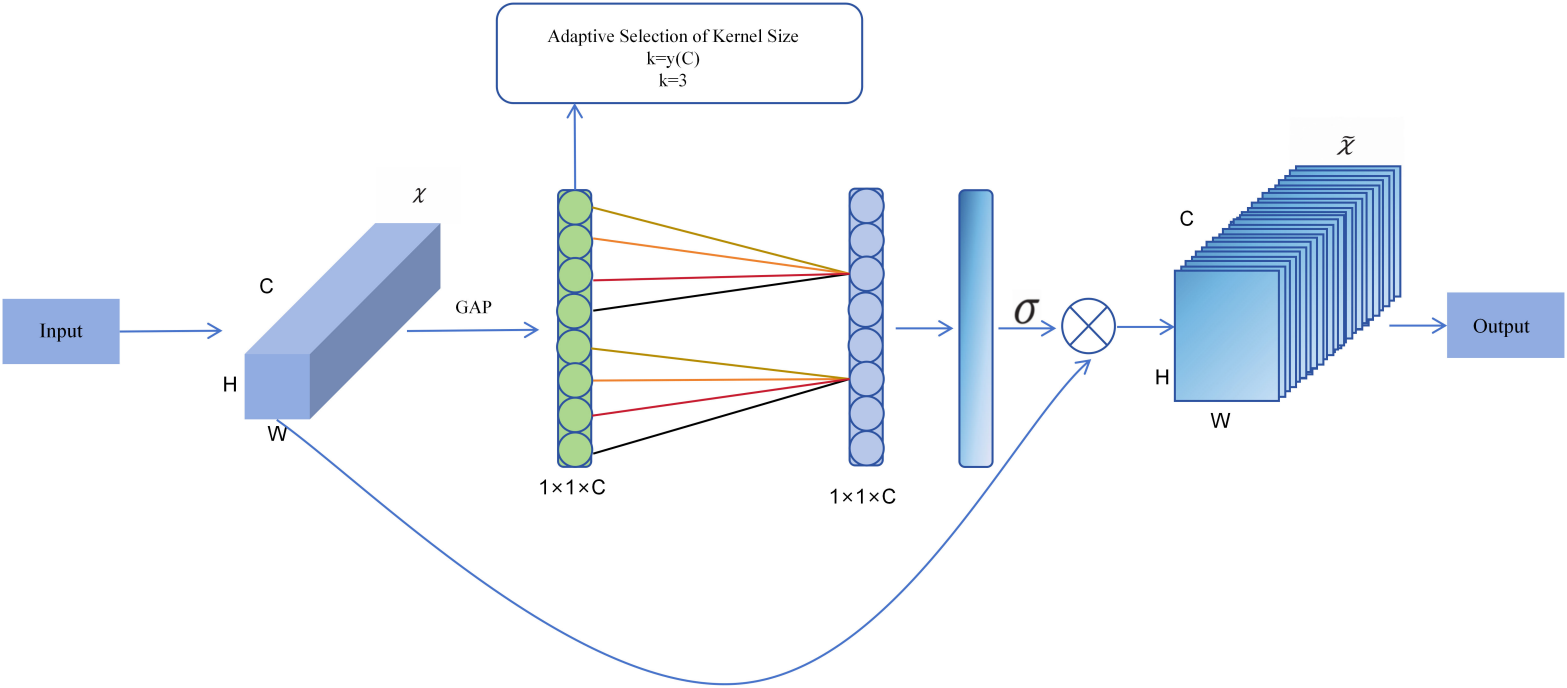
Structure of the efficient channel attention (ECA) module.

MobileNetV3 was used as the encoder backbone because it provides a favorable balance between representation capacity and computational efficiency (Howard et al., 2019). As illustrated in **Figure 4**, AAF and ECA were integrated into each MobileNetV3 bottleneck to enhance nonlinear feature extraction and channel-wise recalibration. The detailed AAF and ECA structures are shown in **Figures 5** and **6**, respectively.

AAF replaces the fixed activation functions after the expansion and depthwise convolutions. Each bottleneck therefore contains two AAF modules, resulting in 30 AAF modules across the backbone. AAF applies parallel ReLU6 and SiLU branches, generates channel-wise fusion weights through global average pooling, two convolutional layers, and a sigmoid function, and combines the two responses by weighted summation.

ECA is inserted after projection batch normalization and before residual addition. Because the backbone contains 15 bottleneck blocks, 15 ECA modules are used. Each ECA module applies global average pooling followed by a one-dimensional convolution with a kernel size of 3, without additional spatial convolutions or channel expansion. Its computational contribution is therefore evaluated using complete-network complexity metrics in **Table 1**.

**Table 1:** Ablation analysis of AAF and ECA modules in the complete segmentation framework.

Variant	Description	mIoU (%)	Precision (%)	BF1 (%)	BIoU (%)	Params (M)	GFLOPs
Decoder-Matched Baseline	OS=16 + MidFusion, w/o AAF and ECA	94.22	96.46	94.01	89.56	14.85	90.37
AAF	AAF only	94.52	96.78	94.50	90.84	19.06	90.42
ECA	ECA only	94.97	96.97	94.15	92.01	14.86	90.38
AAF+ECA	Full model	95.87	97.63	95.88	92.13	19.07	90.43

All variants used the same MobileNetV3 backbone and OS = 16 MidFusion decoder. The Decoder-Matched Baseline contained neither AAF nor ECA and was used as the reference configuration for calculating computational overhead. Only AAF and ECA were varied across configurations.

ReLU6 and SiLU were selected for their complementary response characteristics rather than as universally optimal activations. ReLU6 provides bounded rectification and is widely used in MobileNetV3 architectures (Howard et al., 2019), whereas SiLU provides smooth self-gated activation that preserves low-amplitude signals (Elfwing et al., 2018). GELU and Mish were included as alternative smooth activations in the controlled comparison (Hendrycks and Gimpel, 2016; Misra, 2020). Standard Swish with β = 1 was not evaluated separately because it is mathematically identical to SiLU (Ramachandran et al., 2017; Elfwing et al., 2018). ECA was adopted as a lightweight channel-attention mechanism (Wang et al., 2020) and compared with representative attention alternatives under the fixed AAF configuration.

#### 2.3.3 Customized ASPP module

**Figure 7:**
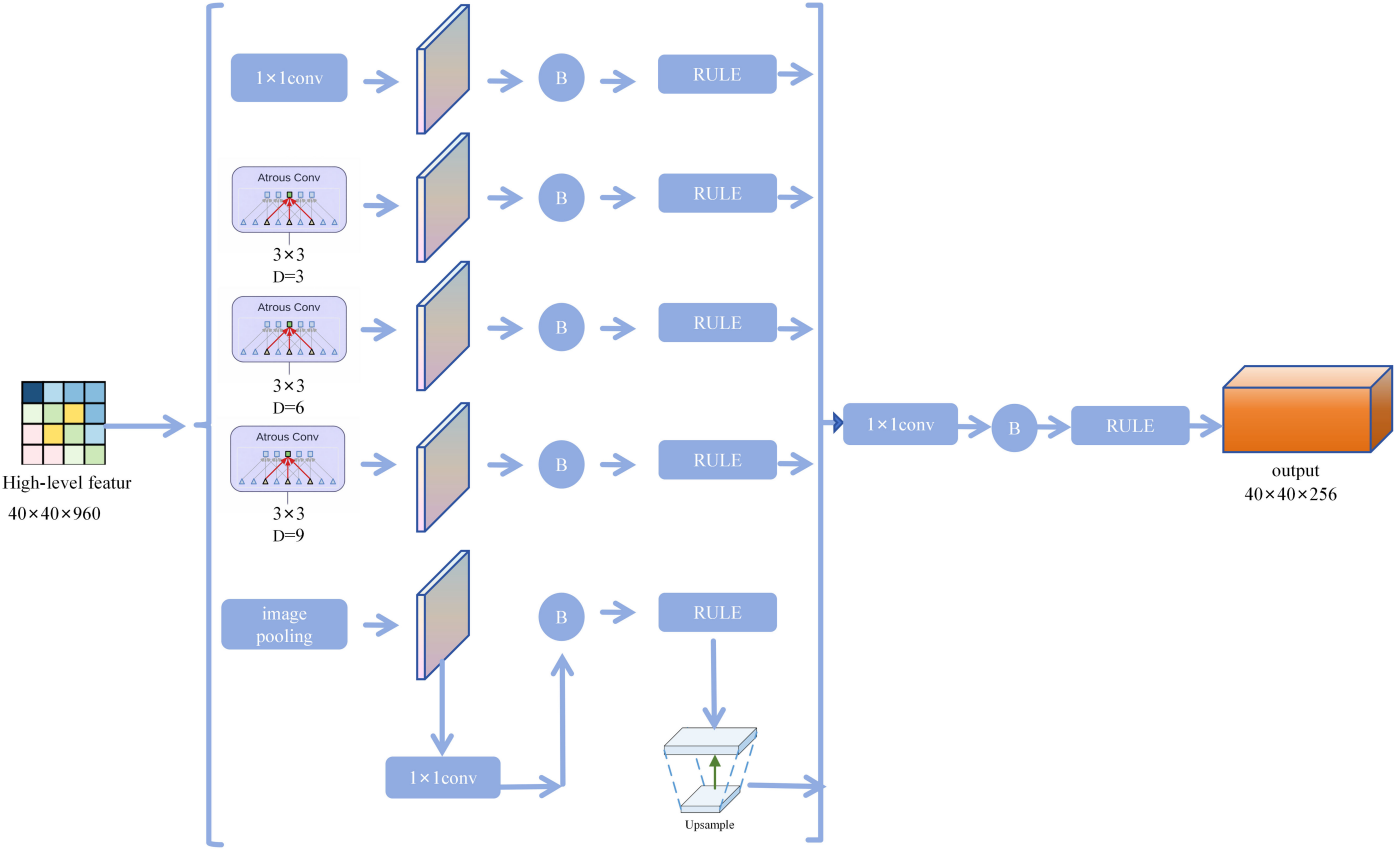
Customized ASPP structure.

The high-level feature map is processed by a customized ASPP module to strengthen multiscale context modeling, as shown in **Figure 7**. The module contains three atrous convolution branches with dilation rates of 3, 6, and 9, together with a 1 × 1 convolution branch and an image-level pooling branch. Their outputs are concatenated and fused through a 1 × 1 convolution block to produce the ASPP representation.

The lower-rate configuration {3, 6, 9} was designed to retain denser spatial sampling for small, fragmented weed regions and crop–weed boundaries while aggregating local-to-intermediate context. Smaller dilation rates may provide insufficient contextual coverage, whereas larger rates increase sampling intervals and may weaken local-detail representation. Because the image-level pooling branch supplies global context, the atrous branches primarily capture local and intermediate spatial information. Alternative dilation-rate configurations were evaluated through the controlled ablation reported in **Table 2**.

**Table 2:** Ablation of ASPP dilation-rate configurations under the fixed AAF + ECA framework.

Dilation rates	mIoU (%)	BF1 (%)	BIoU (%)	Params (M)	GFLOPs
2, 4, 6	94.32	95.11	90.86	19.07	90.43
3, 6, 9	95.87	95.88	92.13	19.07	90.43
6, 12, 18	95.58	95.37	91.98	19.07	90.43

All variants used the same MobileNetV3 backbone, AAF + ECA configuration, ASPP branch number, channel widths, hierarchical decoder, loss function, training protocol, and data split. Only the atrous dilation rates were changed.

#### 2.3.4 Hierarchical decoder with residual refinement

An OS = 16 hierarchical decoder was designed to recover spatial details and boundary information through coarse-to-fine feature fusion. The ASPP output is first upsampled and fused with mid-level features, followed by residual refinement. The refined representation is then upsampled and fused with low-level features before a second residual refinement stage. A classifier head generates segmentation logits, which are upsampled to the input resolution.

This decoder combines high-level semantics with shallow spatial details to improve prediction continuity and boundary recovery for small, irregular, and partially occluded weed regions.

### 2.4 Loss function and training strategy

To optimize difficult weed regions and ambiguous crop–weed boundaries, a hybrid Focal Cross-Entropy–Dice loss was used (Milletari et al., 2016; Lin et al., 2017), as defined in Equation 1:

(1) $$\begin{array}{c} L=0.6LFocalCE+0.4LDice \end{array}$$

where LFocalCE and ​ LDice denote the Focal Cross-Entropy loss and Dice loss, respectively. The focal term emphasizes difficult pixels, whereas the Dice term improves prediction–ground-truth overlap, particularly for small and fragmented weed regions. FocalCE used a focusing parameter of 1.5, class weights of 0.65 for pepper and 4.5 for weed, and label smoothing of 0.05. This task-specific configuration increased attention to difficult weed pixels and stabilized optimization; the class weights were not intended to correct a severe global pixel imbalance.

AdamW was optimized with linear warm-up followed by cosine annealing (Loshchilov and Hutter, 2019). Training used a batch size of 4, 200 epochs, and 640 × 640-pixel inputs; the initial learning rate and weight decay are reported in **Table 3**. Key hyperparameters were selected through staged empirical tuning on the Shanghai validation set rather than an exhaustive grid search. Initial settings followed standard formulations of Focal loss, Dice loss, and AdamW (Milletari et al., 2016; Lin et al., 2017; Loshchilov and Hutter, 2019). The focal parameter, hybrid-loss weights, class weights, and initial learning rate were adjusted sequentially while other settings were fixed, and the final configuration was selected by validation mIoU. Neither the Shanghai internal test set nor the Anhui external test set was used for hyperparameter selection.

**Table 3:** Experimental parameter configuration.

Parameters	Value
CPU	Intel Core i5-14600KF
GPU	NVIDIA RTX 4070 SUPER
Framework	PyTorch 2.5.1
Optimizer	AdamW
Learning rate	$5\times 10^{-4}$
Weight decay	$1\times 10^{-4}$
Training batch size	4
Inference batch size	1
Training epochs	200
Input size	640 × 640
Precision for FPS test	FP32

Note: The common training hyperparameters were applied to all compared models unless otherwise specified.

### 2.5 Experimental setup and comparative design

All experiments were conducted on a workstation equipped with an Intel Core i5-14600KF CPU and an NVIDIA GeForce RTX 4070 SUPER GPU. The software environment included Python 3.9.23, PyTorch 2.5.1, CUDA 11.8, and cuDNN 9.1.0. The detailed experimental parameters are listed in **Table 3**.

The proposed framework was compared with U-Net, PSPNet, SegFormer (Xie et al., 2021), DeepLabV3+–ResNet50, PP-LiteSeg, PIDNet, BiSeNetV2, Fast-SCNN, and DeepLabV3+–MV3, representing classical convolutional, encoder–decoder, Transformer-based, and lightweight DeepLabV3+-based methods. Four recent agricultural models—CWRepViT-Net (Gomroki et al., 2025), EDM-UNet (Gao et al., 2025), KD-Fast-SCNN (Agustian et al., 2026), and PSPEdgeWeedNet (Pai et al., 2025)—were additionally evaluated. These methods and the proposed model were trained on the Shanghai training set, selected solely by Shanghai validation performance, evaluated on the independent Shanghai test set, and then directly tested on the Anhui external set without retraining or fine-tuning.

Ablation experiments assessed AAF, ECA, decoder design, loss function, and class weighting. To isolate AAF and ECA, four variants sharing the OS = 16 MidFusion decoder were compared: the Decoder-Matched Baseline without AAF or ECA, AAF only, ECA only, and AAF+ECA. Decoder effects were evaluated separately using the standard DeepLabV3+ decoder, OS = 16 + MidFusion, and OS = 16 + MidFusion + residual refinement. Loss-function ablation compared CE + Dice with FocalCE + Dice, with the latter following the weighted hybrid strategy in Section 2.4. Class-weight ablation compared the main pepper/weed weights of 0.65/4.5 with relaxed weights of 0.65/2.0. Except for the factor being tested, all training settings were unchanged.

For fair comparison, all baseline models used the same training, validation, and independent test sets, input resolution, batch size, augmentation, weighted sampling, training epochs, optimizer, and learning-rate schedule. Their standard architecture-specific components were retained without additional model-specific modules, and hyperparameter selection and checkpoint selection used only the validation set. U-Net, SegFormer, PIDNet, DeepLabV3+–MobileNetV3, and the proposed model were independently trained with six identical random seeds. For each run, the checkpoint with the highest validation mIoU was evaluated on the Shanghai independent test set. Seed-matched outcomes were compared using two-sided paired t-tests, with Holm–Bonferroni correction across the four representative baseline comparisons; mean ± standard deviation values and adjusted p-values are reported in **Table 4**. Unless otherwise stated, other results were obtained from the validation-selected checkpoint of the corresponding configuration.

**Table 4:** Repeated-run performance and statistical comparison of representative segmentation models over six random seeds on the Shanghai internal independent test set.

Model	mIoU (%)	p_adj vs. Ours	BF1 (%)	p_adj vs. Ours	BIoU (%)	p_adj vs. Ours
U-Net	94.10 ± 0.22	6.0 × 10^-6^	87.35 ± 0.28	1.6 × 10^-9^	83.08 ± 0.31	2.4 × 10^-9^
SegFormer	93.28 ± 0.19	2.7 × 10^-6^	86.22 ± 0.25	1.5 × 10^-9^	80.11 ± 0.29	1.2 × 10^-9^
PIDNet	93.94 ± 0.24	6.0 × 10^-6^	85.62 ± 0.20	8.0 × 10^-10^	83.44 ± 0.23	1.9 × 10^-9^
DLV3+MV3	93.76 ± 0.21	6.0 × 10^-6^	94.03 ± 0.19	2.7 × 10^-6^	89.26 ± 0.22	5.5 × 10^-7^
Ours	95.82± 0.15	—	95.75 ± 0.14	—	92.04 ± 0.18	—

Values are reported as mean ± standard deviation over six independently initialized runs. Each adjusted p-value represents a two-sided paired t-test comparing the corresponding baseline with the proposed model using seed-matched results. Holm–Bonferroni correction was applied across the four representative baseline comparisons separately for each metric (df = 5). Dashes denote the reference model.

For activation selection, ECA and all other components were fixed, and only the AAF activation configuration was varied. For attention comparison, ReLU6–SiLU AAF was fixed and each candidate module replaced ECA at identical locations. SE, CBAM, Coordinate Attention, SimAM, and EMA were included as representative alternatives (Hu et al., 2018; Woo et al., 2018; Hou et al., 2020; Yang et al., 2021; Ouyang et al., 2023).

Parameters and GFLOPs were calculated for the complete network, including the backbone, ASPP module, decoder, and classifier head. GFLOPs were measured in evaluation mode with THOP using a 1 × 3 × 640 × 640 input tensor and its convolution/MAC counting convention. Thus, ablation values represent complete variants rather than isolated modules. Inference speed was measured on the RTX 4070 SUPER using FP32 precision, batch size 1, and 1 × 3 × 640 × 640 inputs. Models were evaluated in model.eval() mode under torch.no_grad(); FPS was calculated from synchronized, warm-up-excluded single-image forward-pass latency, excluding disk I/O, preprocessing, and postprocessing.

### 2.6 Evaluation metrics

Precision, recall, F1-score, intersection over union (IoU), mean intersection over union (mIoU), boundary F1-score (BF1), boundary IoU (BIoU), model parameters, GFLOPs, and FPS were used to evaluate model performance on the independent test set. IoU and mIoU were used to evaluate region-level segmentation accuracy. BF1 and BIoU were introduced to evaluate boundary localization accuracy and boundary-region consistency (Csurka et al., 2013; Cheng et al., 2021). Parameters and GFLOPs were used to measure model complexity, while FPS was used to evaluate inference efficiency. In addition to mean metrics, class-wise precision, recall, IoU, BF1, and BIoU were calculated for pepper and weed to evaluate whether the proposed model achieved balanced region-level and boundary-level performance across both semantic classes.

The conventional segmentation metrics were calculated using Equations 2–6 as follows:

(2) $$\begin{array}{c} Precision=TP/(TP+FP) \end{array}$$

(3) $$\begin{array}{c} Recall=TP/(TP+FN) \end{array}$$

(4) $$\begin{array}{c} F1=(2\times Precision\times Recall)/(Precision+Recall) \end{array}$$

(5) $$\begin{array}{c} IoU=TP/(TP+FP+FN) \end{array}$$

(6) $$\begin{array}{c} mIoU=(1/N)\sum_{i=1}^{N}IoU_{i} \end{array}$$

where Precision and Recall denote pixel-level precision and recall, respectively. TP, FP, and FN denote true-positive, false-positive, and false-negative pixels, respectively. N denotes the number of valid semantic classes, and $IoU_{i}\;$denotes the IoU of the i-th class.

In addition to conventional region-level metrics, BF1 and BIoU were used to evaluate boundary quality. BF1 measures contour matching based on boundary precision and boundary recall, whereas BIoU evaluates boundary-region overlap and is more sensitive to boundary errors than standard IoU.

The boundary map was extracted using Equation 7 as follows:

(7) $$\begin{array}{c} B(M)=M-erode(M,k,d) \end{array}$$

where M denotes the binary segmentation mask, k is a 3×3 structuring element, d is the boundary width, and B(M) denotes the extracted boundary map.

The boundary F1-score and boundary IoU for class c were calculated using Equations 8 and 9 as follows:

(8) $$\begin{array}{c} BF1_{c}=2P_{c}^{b}R_{c}^{b}/(P_{c}^{b}+R_{c}^{b}) \end{array}$$

(9) $$\begin{array}{c} BIoU_{c}=TP_{c}^{b}/(TP_{c}^{b}+FP_{c}^{b}+FN_{c}^{b}) \end{array}$$

where $P_{c}^{b}$ and $R_{c}^{b}$ denote the boundary precision and boundary recall of class c, respectively. $TP_{c}^{b}$, $FP_{c}^{b}$, and $FN_{c}^{b}$ denote boundary-level true-positive, false-positive, and false-negative pixels of class c, respectively. Pixels labeled as 255 were ignored during both loss calculation and metric evaluation.

## 3 Results

### 3.1 Training performance of the proposed model

**Figure 8:**
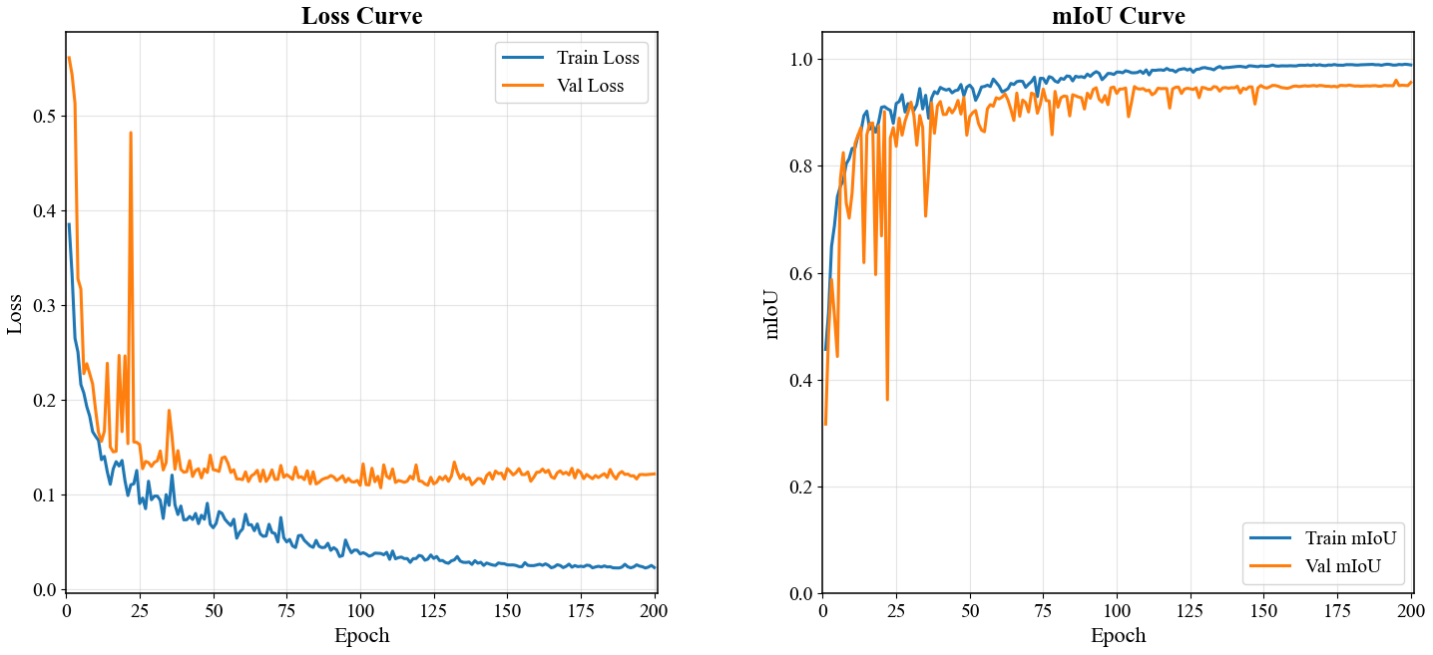
Training loss and mIoU curves of the MobileNetV3-based plain model.

**Figure 9:**
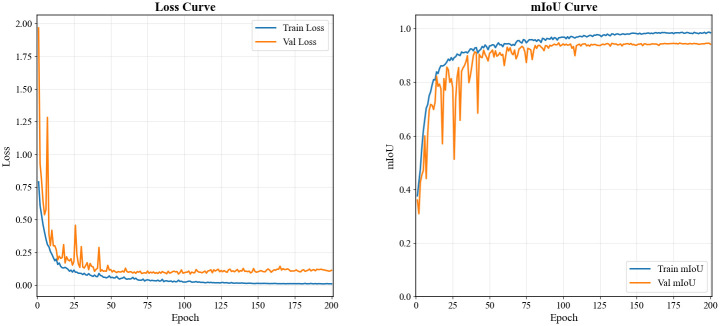
Training loss and mIoU curves of the proposed model.

Training behavior was evaluated by comparing the proposed model with the plain MobileNetV3-based DeepLabV3+ baseline. **Figure 8** presents the training outputs of the plain baseline, whereas **Figure 9** presents the corresponding outputs of the proposed model. Both models converged rapidly during the early training stage. Compared with the plain baseline, the proposed model exhibited a smoother late-stage loss trajectory, maintained lower loss values, and achieved higher mIoU during most training epochs, indicating more stable optimization and improved segmentation learning. The checkpoint with the highest validation mIoU of the proposed model was selected for subsequent evaluation on the independent test set.

**Figure 10:**
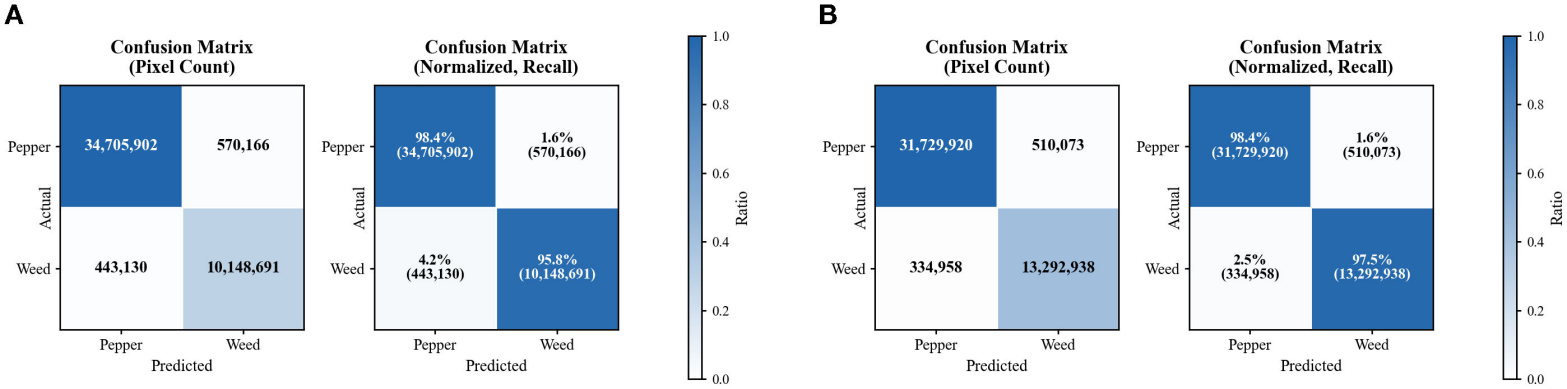
Confusion matrices of the plain model and the proposed model on the pepper-field weed independent test set. **(a)** Plain MobileNetV3-based model; **(b)** proposed model.

**Figure 10** presents the confusion matrices of the Plain baseline and the full AAF+ECA model on the independent test set. Compared with the Plain baseline, the full AAF+ECA model showed stronger diagonal dominance and fewer crop–weed misclassifications. In particular, fewer weed pixels were misclassified as pepper, indicating that the proposed feature enhancement and fusion strategy improved the discrimination of weed regions under complex field conditions.

### 3.2 Comparison with representative segmentation networks

To evaluate the proposed framework, nine representative segmentation models were compared on the independent test set, including U-Net, PSPNet, SegFormer, DeepLabV3+–ResNet50, PP-LiteSeg, PIDNet, BiSeNetV2, Fast-SCNN, and DeepLabV3+–MobileNetV3. These models represent classical convolutional, encoder–decoder, Transformer-based, and lightweight DeepLabV3+-based architectures. The quantitative results are summarized in **Table 5**.

**Table 5:** Performance comparison of different models on pepper-field weed segmentation.

Model	mIoU (%)	Recall (%)	BF1 (%)	BIoU (%)	Params (M)	GFLOPs	FPS
U-Net	94.12	97.17	87.30	83.02	31.42	121.70	28.86
PSPNet	94.15	96.26	87.02	80.33	49.00	144.20	24.63
SegFormer	93.26	96.36	86.17	80.02	3.71	10.58	59.05
DeepLabV3+–ResNet50	95.09	97.24	85.48	82.30	40.35	108.52	26.49
PP-LiteSeg	93.36	96.49	84.32	88.61	6.556	17.637	52.43
PIDNet	94.09	96.11	85.66	83.54	7.622	18.421	51.22
BiSeNetV2	93.21	96.34	85.78	82.33	3.110	38.133	52.35
Fast-SCNN	93.58	96.02	86.97	78.32	1.234	2.635	60.05
DLV3+MV3	93.91	97.10	94.01	89.24	11.72	37.93	48.47
Ours	95.87	97.98	95.88	92.13	19.07	90.43	46.86

As shown in **Table 5**, the proposed model achieved the highest mIoU (95.87%), recall (97.98%), BF1 (95.88%), and BIoU (92.13%) among the compared methods. The simultaneous improvement in mIoU and boundary metrics indicates that the model improved both overall region classification and crop–weed boundary localization. Compared with the DeepLabV3+–MobileNetV3 baseline, mIoU increased from 93.91% to 95.87%, BF1 from 94.01% to 95.88%, and BIoU from 89.24% to 92.13%. These gains indicate that the proposed enhancements improved segmentation continuity and boundary consistency beyond the original lightweight DeepLabV3+–MobileNetV3 configuration.

**Table 4** further evaluates the stability of the main comparative conclusions across six random seeds. The proposed model achieved the highest mean mIoU, BF1, and BIoU, together with the smallest standard deviations across these metrics. The adjusted p-values from the seed-matched paired t-tests remained significant after Holm–Bonferroni correction, indicating that the observed improvements over the representative baselines were not attributable solely to random initialization. Thus, **Table 5** presents the representative test result, whereas **Table 4** confirms that the performance advantage was maintained across repeated training runs.

The efficiency-related results in **Table 5** show that the proposed model required 19.07 M parameters and 90.43 GFLOPs while achieving 46.86 FPS. Fast-SCNN achieved the lowest computational cost and highest inference speed, but its lower mIoU, BF1, and BIoU indicate weaker region recovery and boundary delineation. In contrast, the proposed method outperformed heavier convolutional models, including U-Net, PSPNet, and DeepLabV3+–ResNet50, in mIoU and boundary metrics while maintaining comparable inference speed. These results indicate that the proposed framework achieved a practical balance between segmentation accuracy, boundary quality, and computational efficiency.

**Figure 11:**
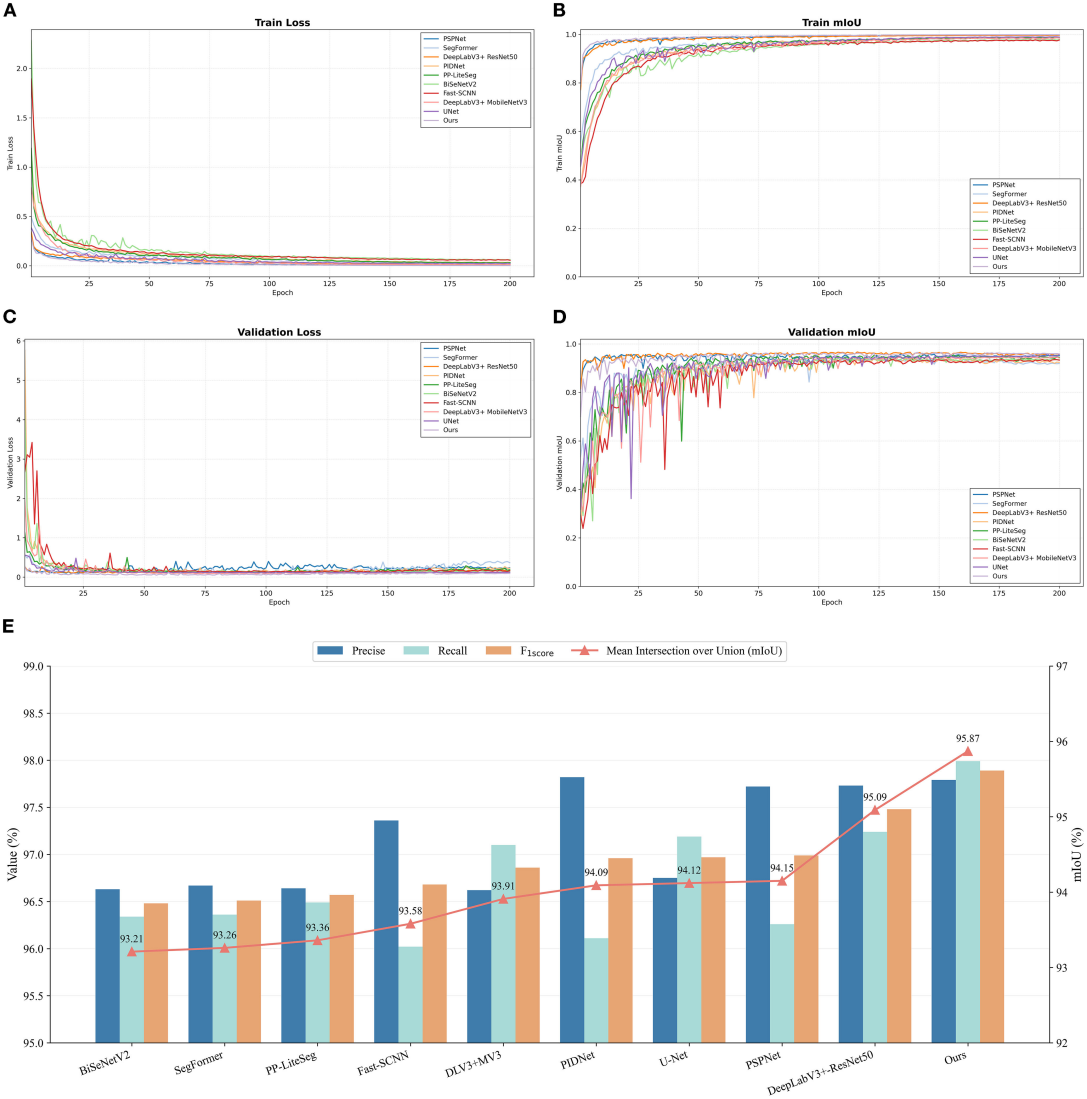
Training behavior and quantitative performance comparison of different models on pepper-field weed segmentation. **(a)** Training loss curves; **(b)** training mIoU curves; **(c)** validation loss curves; **(d)** validation mIoU curves; **(e)** quantitative comparison of mIoU, precision, recall, and F1-score evaluated on the independent test set.

**Figure 11** provides complementary evidence of training behavior and quantitative performance. **Figures 11a, b** show the training loss and training mIoU curves, whereas **Figures 11c, d** present the corresponding validation curves. The proposed model showed more stable late-stage optimization and maintained stronger validation performance than the representative comparison models. **Figure 11e** further summarizes mIoU, precision, recall, and F1-score, visually confirming the quantitative advantage reported in **Table 5**. Differences in validation-loss smoothness reflect model-specific optimization dynamics under the shared training protocol rather than unequal training duration or hyperparameter-search effort.

**Figure 12:**
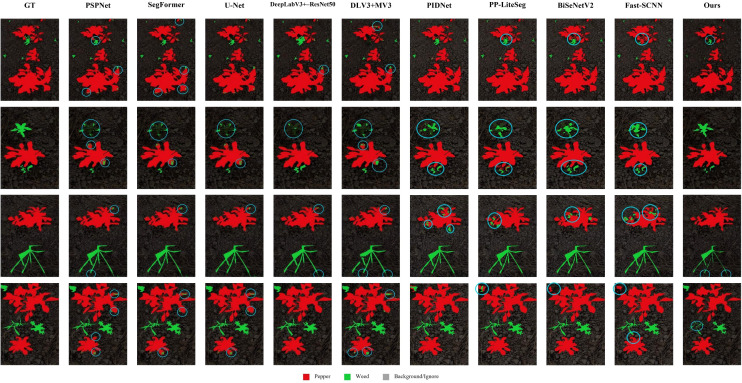
Visual comparison of segmentation results from different models on representative pepper-field weed images from the independent test set. Cyan circles indicate representative regions where segmentation errors were reduced or boundary details were improved.

Representative segmentation examples are shown in **Figure 12**. Compared with the baseline and other representative models, the proposed method produced more continuous weed regions, reduced fragmented predictions, and generated clearer crop–weed boundaries. These visual results are consistent with the higher BF1 and BIoU values in **Table 5**, indicating improved boundary delineation in addition to higher region-level accuracy.

**Table 6** reports the class-wise segmentation and boundary metrics. For pepper, the proposed model achieved 97.65% IoU, 97.35% BF1, 94.83% BIoU, 99.00% recall, and 98.62% precision. For weed, it achieved 94.09% IoU, 94.42% BF1, 89.43% BIoU, 96.96% recall, and 96.64% precision. Although weed performance was slightly lower than pepper performance, which is consistent with the smaller, fragmented, and partially occluded characteristics of weed regions, all weed metrics remained high. The mean values of IoU, BF1, BIoU, recall, and precision were 95.87%, 95.88%, 92.13%, 97.98%, and 97.63%, respectively, confirming balanced region-level and boundary-level performance across both semantic classes.

**Table 6:** Per-class segmentation and boundary metrics of the proposed model on the independent test set.

Class	IoU (%)	BF1 (%)	BIoU (%)	Recall(%)	Precision(%)
Pepper	97.65	97.35	94.83	99.00	98.62
Weed	94.09	94.42	89.43	96.96	96.64
Mean	95.87	95.88	92.13	97.98	97.63

### 3.3 Ablation study

To evaluate the contribution of each component in the proposed framework, ablation experiments were conducted from four aspects: the AAF and ECA modules, decoder design, loss function, and class-weight configuration.

#### 3.3.1 Effects of the OS = 16 MidFusion decoder, AAF, and ECA

Four network configurations with the same OS = 16 MidFusion decoder were compared to evaluate the individual and combined effects of AAF and ECA, as summarized in **Table 1**. The Decoder-Matched Baseline, which contained neither AAF nor ECA, achieved 94.22% mIoU and 89.56% BIoU and was used as the reference configuration.

Under the same OS = 16 MidFusion decoder configuration, the AAF-only model increased mIoU from 94.22% to 94.52% and BIoU from 89.56% to 90.84%. The ECA-only model achieved 94.97% mIoU and 92.01% BIoU. The AAF+ECA model achieved the best overall performance, with 95.87% mIoU, 97.63% precision, 95.88% BF1, and 92.13% BIoU. These results indicate that AAF and ECA provided complementary improvements beyond the Decoder-Matched Baseline.

Relative to the Decoder-Matched Baseline, AAF increased the parameter count by 4.21 M and the computational cost by 0.05 GFLOPs, yielding a 0.30-percentage-point gain in mIoU. ECA introduced only 0.01 M additional parameters and 0.01 GFLOPs, while improving mIoU by 0.75 percentage points. The combined AAF + ECA configuration increased the total complexity by 4.22 M parameters and 0.06 GFLOPs, while improving mIoU by 1.65 percentage points. The corresponding parameter and GFLOPs values are reported in **Table 1**. These results show that ECA introduced negligible computational overhead, whereas AAF accounted for most of the additional parameters while providing complementary feature-representation gains.

**Figure 13:**
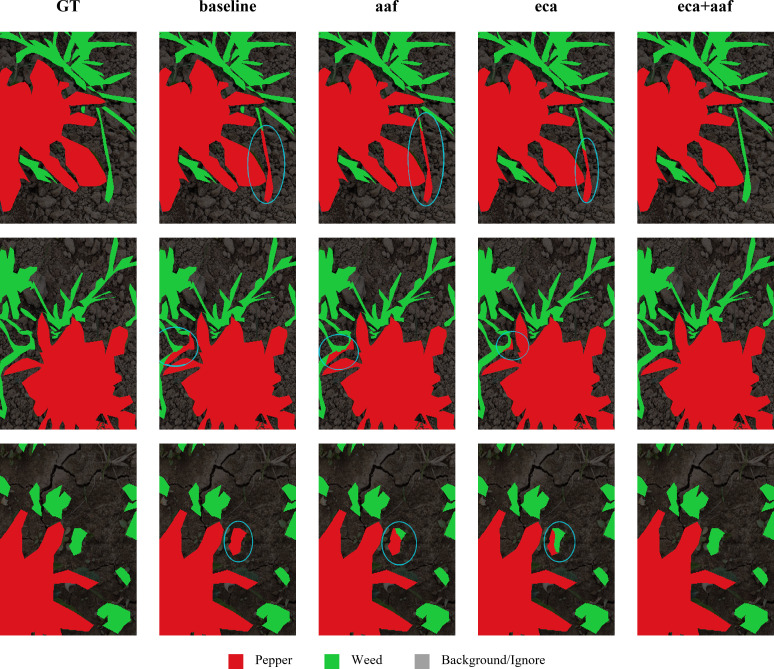
Visual comparison of selected ablation variants on representative pepper-field weed test images. GT denotes the ground truth. Plain, AAF, ECA, and AAF+ECA denote the selected configurations shown in this figure. Quantitative results for all five configurations, including the Decoder-Matched Baseline, are reported in **Table 1**. Cyan circles highlight representative segmentation differences among the displayed variants.

The visual ablation results are shown in **Figure 13**.

#### 3.3.2 Effect of decoder variants

Three decoder variants were compared to evaluate the proposed decoder design: the standard DeepLabV3+ decoder, OS = 16 + MidFusion, and OS = 16 + MidFusion + residual refinement. The results are shown in **Table 7**.

**Table 7:** Comparison of decoder variants.

Variant	Description	Best mIoU (%)		Precision (%)	Recall (%)	F1-score (%)
D1	Standard DeepLabV3+ decoder	94.42		97.39	97.65	97.52
D2	OS=16 + MidFusion	95.24		97.28	98.10	97.69
D3	OS=16 + MidFusion + residual refinement	95.87		97.63	97.98	97.83

As shown in **Table 7**, the standard DeepLabV3+ decoder achieved 94.42% mIoU. After adding OS = 16 + MidFusion, the mIoU increased to 95.24%, indicating that mid-level feature fusion improved the integration of semantic and spatial information. With the further addition of residual refinement, the D3 variant achieved the best performance, with 95.87% mIoU, 97.63% precision, 97.98% recall, and 97.83% F1-score. These results suggest that multi-level feature fusion and residual refinement improved overall segmentation performance and spatial-detail recovery.

#### 3.3.3 Effect of loss functions

To evaluate the influence of the optimization objective, CE + Dice and FocalCE + Dice were compared, as summarized in **Table 8**. FocalCE + Dice achieved the best overall performance, with an mIoU of 95.87%, precision of 97.63%, recall of 97.98%, and F1-score of 97.83%. Compared with CE + Dice, FocalCE + Dice improved mIoU from 95.12% to 95.87% and recall from 97.52% to 97.98%. These results indicate that the focal term improved learning for difficult pixels, whereas Dice loss enhanced region-level overlap for small and spatially fragmented weed regions. Therefore, FocalCE + Dice was selected as the final loss configuration.

**Table 8:** Comparison of different loss functions.

Variant	Loss type	Best mIoU (%)	Precision (%)	Recall (%)	F1-score (%)
L1	CE + Dice	95.12	97.92	97.52	97.72
L2	FocalCE + Dice	95.87	97.63	97.98	97.83

#### 3.3.4 Effect of class-weight configuration

The main class-weight configuration was compared with a relaxed class-weight configuration to evaluate the effect of pepper–weed class weighting on segmentation performance. The results are reported in **Table 9**.

**Table 9:** Effect of class-weight configuration on pepper-field weed segmentation.

Version	Best mIoU (%)	Precision (%)	Recall (%)	F1-score (%)
Main class-weight configuration	95.87	97.63	97.98	97.83
Relaxed class-weight configuration	94.29	96.55	97.34	96.94

As shown in **Table 9**, the main class-weight configuration achieved higher values for all reported metrics than the relaxed class-weight configuration. Specifically, mIoU increased from 94.29% to 95.87%, precision increased from 96.55% to 97.63%, recall increased from 97.34% to 97.98%, and F1-score increased from 96.94% to 97.83%. These results indicate that the selected class-weight configuration achieved better overall segmentation performance on the independent test set. Given the moderate global pepper-to-weed pixel ratio, this improvement should be interpreted as the effect of task-specific weighting for difficult weed-region recovery rather than evidence of a severe dataset-level disparity in pixel proportions.

Overall, the ablation results indicate that the final performance was obtained through the combined effects of AAF, ECA, hierarchical decoding, the hybrid loss function, and the class-weight configuration.

### 3.4 Extended validation and design selection

**Figure 14:**
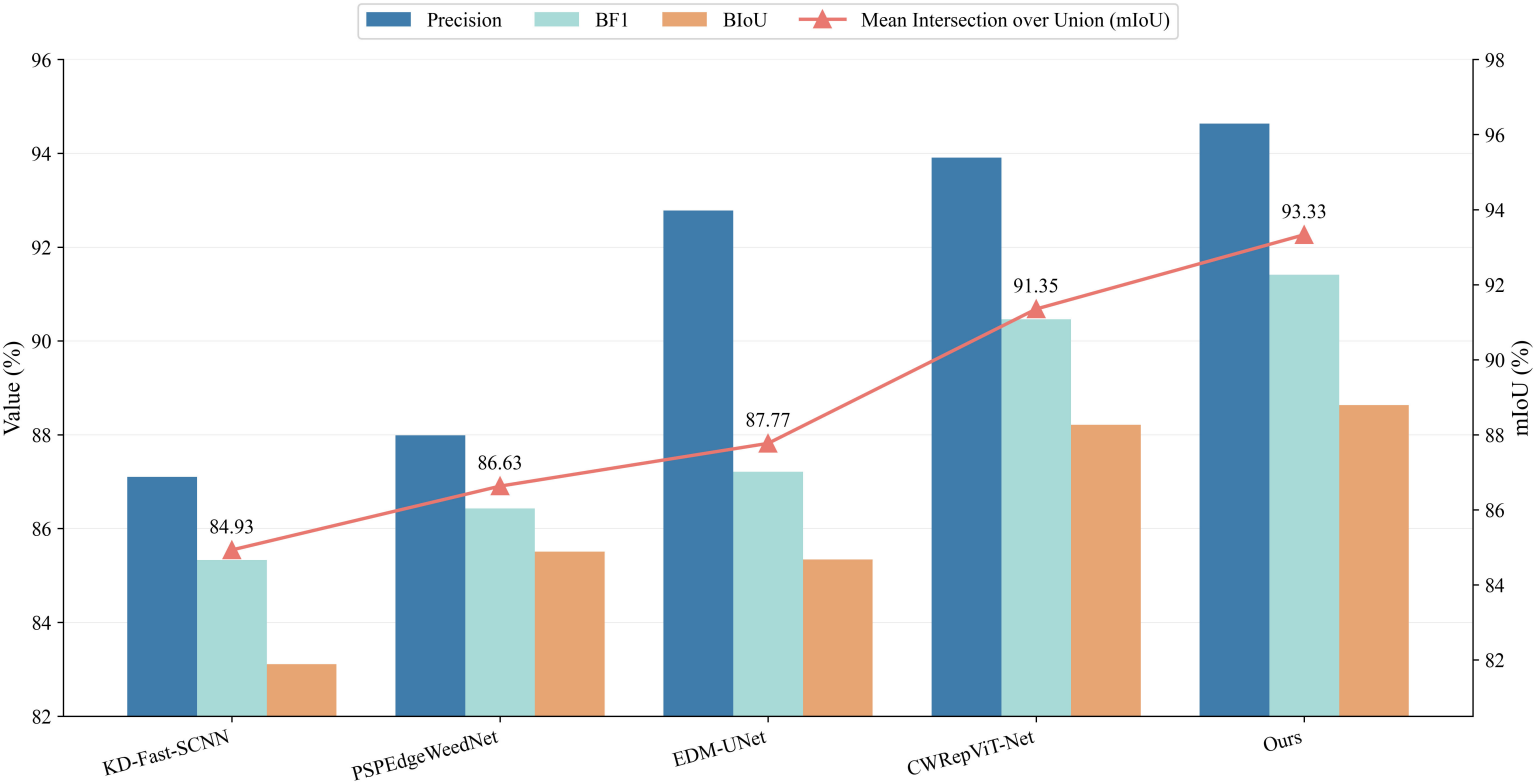
Cross-location quantitative comparison of the proposed method and recent agricultural semantic segmentation models on the external Chizhou, Anhui test set.

**Figure 15:**
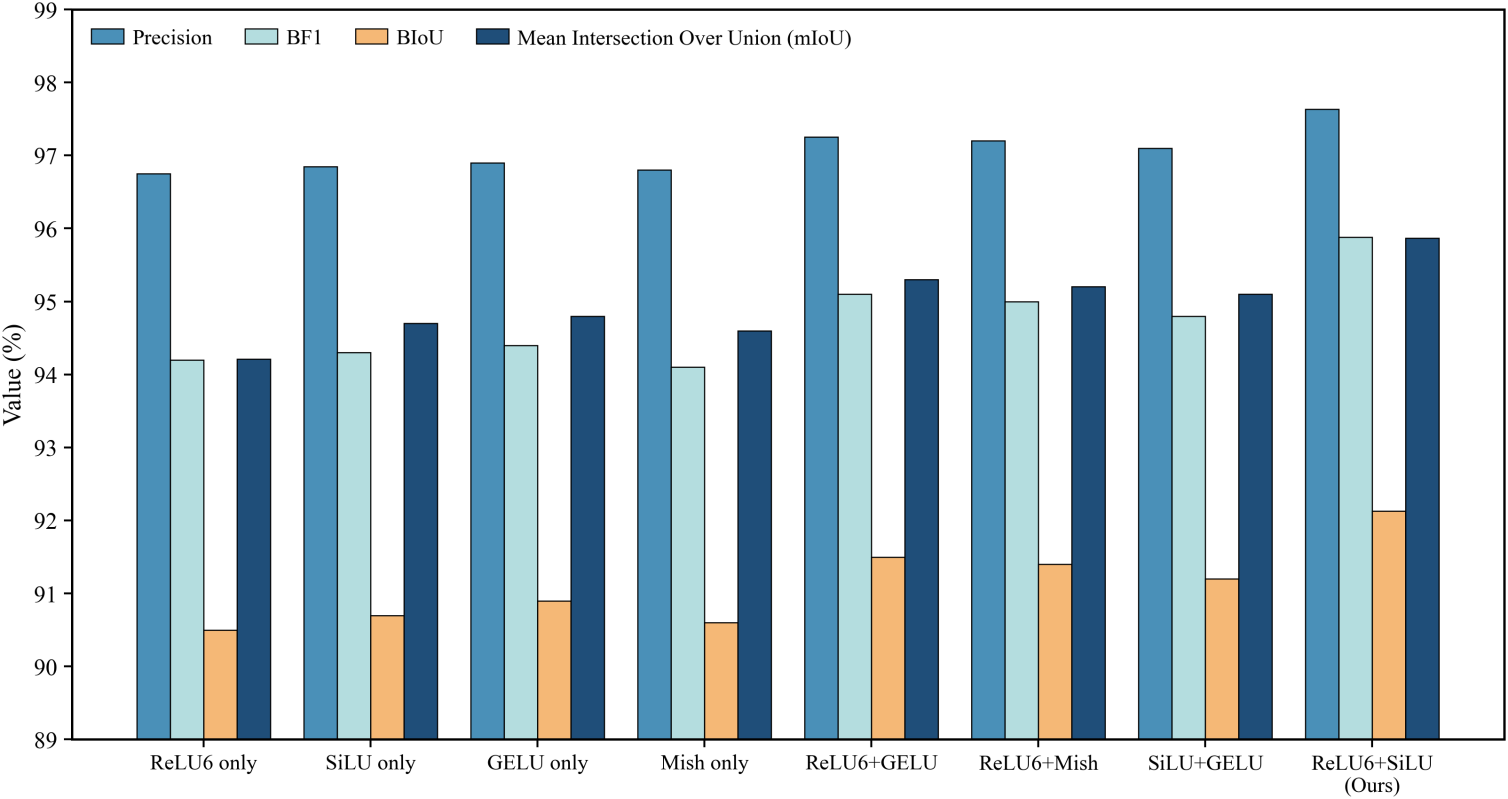
Comparison of activation configurations in the AAF module on the Shanghai internal independent test set.

To extend the comparison beyond the mainstream baselines, the proposed method was evaluated against four recent agricultural segmentation models. **Table 10** shows that the proposed method achieved the highest mIoU, Precision, BF1, and BIoU on the Shanghai internal independent test set. Compared with CWRepViT-Net, the strongest recent agricultural baseline, the proposed method improved mIoU, Precision, BF1, and BIoU by 2.52, 1.72, 3.12, and 1.92 percentage points, respectively. The same five Shanghai-trained models were then directly tested on the geographically distinct Anhui dataset. **Figure 14** shows that the proposed method again achieved the best overall performance, including an external mIoU of 93.33%. The reduction relative to the Shanghai internal result reflects the expected distribution shift between sites, while the retained performance provides additional evidence of cross-location generalization. **Table 2** compares three ASPP dilation-rate configurations under the fixed AAF + ECA framework. The {3, 6, 9} configuration achieved the best overall performance, with 95.87% mIoU, 95.88% BF1, and 92.13% BIoU. Compared with the smaller {2, 4, 6} configuration, it improved mIoU, BF1, and BIoU by 1.55, 0.77, and 1.27 percentage points, respectively. Although the larger {6, 12, 18} configuration improved contextual coverage, it remained lower than {3, 6, 9} by 0.29 percentage points in mIoU, 0.51 percentage points in BF1, and 0.15 percentage points in BIoU. Because all variants had identical parameter counts and GFLOPs, the performance differences can be attributed to the dilation-rate configuration rather than model capacity or computational complexity. These results indicate that {3, 6, 9} provided the most effective balance between local-detail preservation and multiscale contextual aggregation for the present pepper-field dataset. **Figure 15** compares alternative activation configurations within AAF. The single-activation variants used fixed activations, whereas the dual-activation variants employed the same learnable fusion mechanism. ReLU6–SiLU achieved the strongest overall region-level and boundary-level performance among the evaluated activation settings. **Table 11** compares attention mechanisms under the fixed ReLU6–SiLU AAF configuration. AAF + ECA achieved the highest mIoU, BF1, and BIoU while requiring fewer parameters, lower computational cost, and higher inference speed than CBAM and EMA. These controlled comparisons provide empirical support for the selected ReLU6–SiLU activation pair and ECA attention mechanism.

**Table 10:** Comparison of the proposed method with recent agricultural semantic segmentation models on the Shanghai internal independent test set.

Model	mIoU (%)	Precision (%)	BF1 (%)	BIoU (%)
CWRepViT-Net	93. 35	95.91	92.76	90.21
EDM-UNet	89.77	93.78	89.21	87.34
KD-Fast-SCNN	86.93	88.10	87.33	86.11
PSPEdgeWeedNet	88.63	89.99	88.43	87.51
Ours	95.87	97.63	95.88	92.13

**Table 11:** Comparison of attention mechanisms under the fixed AAF configuration.

Variant	Attention module	mIoU (%)	BF1 (%)	BIoU (%)	Params (M)	GFLOPs	FPS
B0	None (AAF-only baseline)	94.52	94.50	90.84	19.06	90.42	48.10
B1	SE	95.35	95.20	91.35	19.86	90.68	44.20
B2	CBAM	95.52	95.38	91.60	20.05	91.05	41.50
B3	Coordinate Attention	95.45	95.30	91.48	19.82	90.75	43.60
B4	SimAM	95.10	94.95	91.10	19.63	90.44	46.50
B5	EMA	95.58	95.42	91.68	19.95	90.90	42.80
B6	ECA (Ours)	95.87	95.88	92.13	19.07	90.43	46.86

The ReLU6–SiLU AAF configuration was fixed for all variants. In B1–B6, the respective attention module was inserted at identical network locations. B0 denotes the AAF-only baseline without attention, and B6 denotes the complete AAF + ECA configuration. All variants were trained and evaluated under the same protocol.

## 4 Discussion

The independent-test results in **Table 5** show that the proposed DeepLabV3+-based framework achieved 95.87% mIoU, 97.98% recall, 95.88% BF1, 92.13% BIoU, and 46.86 FPS with 19.07 M parameters. This combination of region-level accuracy, boundary quality, and inference efficiency is relevant to precision weeding, where inaccurate crop–weed boundaries can cause crop injury, incomplete weed removal, or imprecise herbicide application. The task is particularly challenging in pepper fields because small weeds may occur near stems or beneath overlapping low-canopy leaves.

The performance gains are consistent with the complementary roles of feature enhancement and multi-level fusion. AAF improves nonlinear feature representation, whereas ECA recalibrates channel responses to emphasize informative features and suppress interference. Customized ASPP aggregates multiscale context, and the hierarchical decoder combines deep semantics with mid- and low-level spatial details to improve recovery of small weed regions and crop–weed boundaries. Thus, the contribution is not the use of attention alone, but the coordinated integration of activation-level enhancement, channel recalibration, contextual aggregation, hierarchical decoding, and targeted training for difficult weed regions. Relative to recent lightweight and feature-fusion crop–weed methods, including Janneh et al. (2023) and Fu et al. (2025), the present design specifically addresses the reduced representation capacity of a MobileNetV3 backbone while retaining efficient inference.

The comparison results also clarify the accuracy–efficiency trade-off. Some lightweight models achieved higher speed or lower complexity but lower mIoU, BF1, and BIoU, indicating weaker recovery of small weeds and ambiguous boundaries. Transformer-based models provide broader contextual modeling but may be constrained by dataset scale, training stability, and computational demand. In this setting, the enhanced lightweight convolutional design provided a more balanced solution by preserving efficient inference while improving local discrimination and boundary recovery. This interpretation is consistent with mobile weed-recognition research emphasizing accuracy together with latency, memory use, and hardware-tier adaptability (Sanches et al., 2026).

The ablation results support the contribution of the proposed components and training strategy. FocalCE + Dice improved learning of difficult pixels and overlap for small, fragmented weed regions, while the selected class weights improved overall segmentation performance. Because the global pepper-to-weed ratio was only moderately imbalanced, the sampling, cropping, loss, and weighting strategies should be viewed primarily as methods for difficult weed-region recovery rather than as corrections for severe global class imbalance. **Table 6** further shows strong class-wise performance for both pepper and weed. Although weed metrics were lower than pepper metrics, consistent with the smaller, fragmented, and partially occluded nature of weed regions, they remained high, indicating that the mean results did not conceal a major class-specific weakness.

Several limitations should be noted. The external evaluation was conducted at only one Anhui location, and mIoU decreased from 95.87% on the Shanghai internal test set to 93.33%, indicating cross-location distribution shift, although the proposed method retained the best overall performance. Public-dataset validation was not conducted because pepper-specific benchmark datasets are scarce. In addition, weeds were treated as one semantic class, which supports crop–weed separation but not species-level discrimination. The framework can be extended to multi-class segmentation by replacing the binary classifier head and retraining with species-level annotations. Future work should include more locations, seasons, cultivars, weed communities, imaging conditions, public agricultural datasets when suitable, and embedded-platform evaluation through model compression and hardware-aware optimization.

## 5 Conclusions

This study proposed an improved DeepLabV3+-based semantic segmentation framework for pepper-field weed segmentation under natural field conditions. By combining MobileNetV3, Adaptive Activation Fusion, Efficient Channel Attention, customized multiscale context aggregation, and a hierarchical decoder with residual refinement, the framework was designed to improve feature discrimination, spatial detail recovery, and crop–weed boundary localization. Experimental results on the independent test set showed that the proposed model achieved 95.87% mIoU, 97.98% recall, 95.88% BF1, 92.13% BIoU, and 46.86 FPS. Comparative experiments demonstrated that the proposed framework achieved a better balance between segmentation accuracy, boundary quality, and inference efficiency than representative mainstream, lightweight, and Transformer-based models. The ablation studies further confirmed that AAF, ECA, hierarchical decoding, FocalCE + Dice loss, and the main class-weight configuration jointly contributed to the final performance.

Beyond improving quantitative metrics, this study provides a practical reference for accurate and efficient field perception in pepper-field weed management. Improved recovery of small weed regions and clearer crop–weed boundaries can support more precise spraying, mechanical weeding, and robotic field operations. Nevertheless, broader validation is still needed before large-scale deployment. Future work will expand the evaluation to additional locations, seasons, cultivars, weed communities, and imaging conditions; assess suitable public agricultural datasets when available; and examine deployment on embedded platforms through model compression and hardware-aware optimization.

## Data Availability

The raw data supporting the conclusions of this article will be made available by the authors, without undue reservation.
